# Synchrotron X-ray powder diffraction under high pressures up to 33 MPa for mechanoresponsive materials

**DOI:** 10.1107/S160057752300108X

**Published:** 2023-03-10

**Authors:** Hidetaka Kasai, Jianqiao Liu, Chao-Nan Xu, Eiji Nishibori

**Affiliations:** a Department of Physics, Faculty of Pure and Applied Sciences and Tsukuba Research Center for Energy Materials Science (TREMS), University of Tsukuba, 1-1-1 Tennodai, Tsukuba, Ibaraki 305-8571, Japan; b National Institute of Advanced Industrial Science and Technology (AIST), 807-1 Shuku-machi, Tosu, Saga 841-0052, Japan; c Department of Molecular and Material Sciences, Kyushu University, 6-1 Kasugakoen, Kasuga, Fukuoka 816-8580, Japan; ESRF and Université Grenoble Alpes, France

**Keywords:** X-ray diffraction, synchrotron radiation, high pressure, mechanoresponsive materials

## Abstract

Equipment for synchrotron X-ray diffraction at high pressures up to 33 MPa with an accuracy of ±0.1 MPa using a liquid as a pressure-transmitting medium has been developed. This equipment enables atomic-scale observation of the structural change of mechanoresponsive materials under applied pressures. The validity of the equipment is demonstrated by observation of the pressure dependence of the lattice parameters of copper. The observed bulk modulus of copper was found to be 139 (13) GPa which is a good agreement with the literature value. The developed equipment was subsequently applied to a repeatable mechanoluminescence material, Li_0.12_Na_0.88_NbO_3_:Pr^3+^. The bulk modulus and compressibility along the *a* and *c* axes were determined as 79 (9) GPa, 0.0048 (6) GPa^−1^ and 0.0030 (9) GPa^−1^, respectively, for the *R*3*c* phase. The advance of high-pressure X-ray diffraction will play an important role in understanding mechanoresponsive materials towards their atomic-scale design.

## Abstract 2

Equipment for synchrotron X-ray diffraction at high pressures up to 33 MPa with an accuracy of ±0.1 MPa has been developed for observation of pressure-induced structural changes of mechanoresponsive materials.

## 1. Introduction

Mechanoresponsive materials change their properties by mechanical force in a controlled manner and have attracted considerable attention as one of the smart materials (Weder, 2011 ▸; Kato *et al.*, 2019 ▸). Mechanoresponsive materials have realized a variety of sustainable and wireless applications in engineering and medicine (Weder, 2011 ▸; Wang *et al.*, 2017 ▸; Feng & Smet, 2018 ▸; Zhang *et al.*, 2019 ▸; Xiong *et al.*, 2021 ▸; Chen *et al.*, 2021 ▸). Among them, mechanoluminescence (ML) materials, from which the emission of light is produced by mechanical force, have been widely studied for device applications such as stress sensing (Xu *et al.*, 1999*a* ▸,*b* ▸; Tu *et al.*, 2017 ▸, 2020 ▸), invisible crack visualization (Fujio *et al.*, 2020 ▸), wind-driven display (Jeong *et al.*, 2014 ▸), smart skin (Qian *et al.*, 2018 ▸; Zhao *et al.*, 2021 ▸), pressure memory (Petit *et al.*, 2019 ▸) and optogenetics (Wu *et al.*, 2019 ▸; Zhang *et al.*, 2021 ▸) since the discovery of repeatable ML from ZnS:Mn^2+^ and SrAl_2_O_4_:Er^2+^ by Xu *et al.* (1999*a* ▸,*b* ▸). Such mechanoresponsive applications utilize a mechanical force produced by a finger, by handwriting, in a living body, and in a manufacturing line *etc*., which mostly corresponds to the pressure range from atmospheric pressure to a few tens of MPa, with a sensitivity of, for example, 1 kPa (Tu *et al.*, 2017 ▸). Understanding mechanoresponsive mechanisms on the atomic scale in this pressure range has become increasingly important towards the atomic-scale design of mechanoresponsive materials (Hara *et al.*, 2020 ▸).

Repeatable mechanoresponsive functions depend on an atomic-scale structural change in the elastic deformation of mechanoresponsive materials. This was indicated by the fact that the ML intensity correlates well with applied pressure up to a few tens of MPa with an accuracy of approximately 1/100 of the range for repeatable ML materials (Tu *et al.*, 2017 ▸, 2020 ▸; Yang *et al.*, 2021 ▸). Atomic-scale observation of the elastic deformation is necessary for understanding the mechano­responsive mechanism on the atomic scale.

To observe the structural change of repeatable mechano­responsive materials, structure observation with application of a mechanical force, *i.e.* high pressure, is necessary. Synchrotron radiation is a powerful tool for small samples in an anvil press and in high-pressure cells such as a diamond anvil cells due to its transmittance, high intensity and directivity. The combination of synchrotron radiation and high-pressure techniques is one of the most important advances in high-pressure science (Mao *et al.*, 2016 ▸; McMahon, 2022 ▸). X-ray diffraction under high pressures has been carried out at synchrotron radiation facilities to observe pressure-dependent structure on the atomic scale in the fields of physics, chemistry, materials science, earth and planetary science, *etc*.

The target pressure range for mechanoresponsive materials requires a technique for X-ray diffraction under high pressures up to a few tens of MPa with an accuracy of a few hundred kPa. This pressure range has received little attention in comparison with pressure ranges of more than 100 MPa in high-pressure science. Hansen *et al.* (2015 ▸) reported environments and techniques for powder X-ray diffraction (PXRD) under gas pressures up to 100 MPa. Synchrotron radiation PXRD (SR-PXRD) with high-pressure techniques enabled identification of materials in reactions under high hydrogen pressures (Møller *et al.*, 2014 ▸; Hansen *et al.*, 2015 ▸). So far, PXRD techniques under high pressures from atmospheric pressure up to 100 MPa have focused not on elastic deformation but on more drastic changes such as chemical reactions, gas absorption/desorption and phase transitions. In comparison with such drastic structural changes, it is challenging to observe structural changes in elastic deformation of mechanoresponsive materials due to much smaller changes on the atomic scale.

In the present study, we have developed equipment for SR-PXRD under hydro­static pressures up to 33 MPa using liquid as a pressure-transmitting medium. The equipment is based on our developed reactor for *in situ* SR-PXRD of hydro­thermal synthesis (Fujita *et al.*, 2019 ▸). A pressure of a few tens of MPa is widely used for hydro­thermal synthesis. We have demonstrated the developed equipment by observing the pressure dependence of the lattice parameters of copper. Diffraction data were collected with the equipment installed on the diffractometer at beamline BL02B2 at SPring-8, Japan (Nishibori *et al.*, 2001 ▸; Kawaguchi *et al.*, 2017 ▸). The data allowed us to determine the pressure-dependent lattice parameters with an accuracy of ±2 × 10^−5^ Å, which is comparable with those for general measurements under atmospheric pressure at the beamline. The used X-ray energy of ∼24 keV gives sufficiently high X-ray transmission through the high-pressure container. An exposure time of only several minutes is required to measure the SR-PXRD data with the aforementioned quality. The short measurement time is useful for maintaining stable pressure with an accuracy of ±0.1 MPa during the measurements. We subsequently applied the equipment to a repeatable ML material Li_1–*x*_Na_*x*_NbO_3_:Pr^3+^ (Hara *et al.*, 2020 ▸; Yang *et al.*, 2021 ▸).

## 2. Experimental

### 2.1. Design of the high-pressure equipment

**Figure 1:**
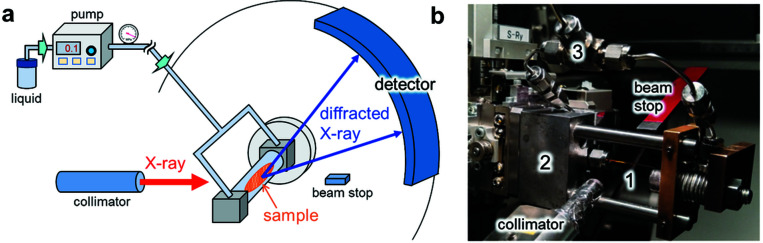
High-pressure equipment for PXRD measurements. (*a*) Overall schematic illustration. (*b*) Environment around a capillary in which a powder sample is loaded. (1) fused silica capillary, (2) holder, (3) stainless steel tube and connector. The holder is attached to the diffractometer at SPring-8 BL02B2.

Figs. 1 ▸(*a*) and 1(*b*) show the high-pressure equipment at beamline BL02B2 at SPring-8 where a large Debye–Scherrer camera is installed (Nishibori *et al.*, 2001 ▸; Kawaguchi *et al.*, 2017 ▸). A powder sample in a fused silica capillary is pressurized by a liquid pressure-transmitting medium using a pump. The stage of the diffractometer enables sample position adjustment and sample oscillation. Fig. 1 ▸(*a*) shows that the pump is placed outside of the experimental hutch to maintain the pressure during PXRD measurements. The capillary is connected to the pump via a stainless steel tube. A pressure gauge, pressure relief valve and manual valve are attached for monitoring the pressure, overpressure protection and manually reducing pressure, respectively.

We modified the *in situ* hydro­thermal reactor (Fujita *et al.*, 2019 ▸) for atomic-scale observation of mechanoresponsive powder samples under high pressures. To apply pressure to the sample equally from both sides, the two lines from the open ends of the capillary are connected to a T-shaped connector with almost the same length and angle, as shown in Fig. 1 ▸(*b*). This prevents sample from flowing by applying pressure. Three copper wires of diameter 0.16 mm and length ∼12 mm are inserted as stoppers at both ends of the fused silica capillary with inner diameter of 0.53 mm, wall thickness of 0.075 mm and length of 50 mm. In Fig. 1 ▸(*b*), the length of the capillary seen between the nuts is approximately 22 mm. The inner part from the T-shaped connector to the capillary is filled with liquid pressure medium using a micropipette. The tube from the pump is also filled with the liquid by pumping and is connected to the T-shaped connector. The pump (Nihon Seimitsu Kagaku NP-KX-500) was used with a flow rate of 0.1 or 0.2 ml min^−1^. The maximum pressure is limited to the maximum discharge pressure of the pump, 35 MPa, in this equipment. The pressure is monitored using a pressure gauge (Krone KDM30). The recorded pressure is shown in Fig. S1 of the supporting information. The pressures are controlled within approximately ±0.1 MPa.

The position of the capillary is monitored using CCD cameras at the beamline during changing of pressure and taking measurements. Initially, the position of the capillary is adjusted using an auto-centering system of the beamline with an accuracy of 0.005 mm. The position of the capillary is modified to the center position every time the pressure is changed before the diffraction measurements are taken. The position shift when changing pressure was typically less than 0.01 mm.

### 2.2. SR-PXRD under high pressures

We measured SR-PXRD data of copper (Kojundo Chemical Lab., 99.99% purity) and the ML material Li_0.12_Na_0.88_NbO_3_:Pr^3+^ (LN12) (Hara *et al.*, 2020 ▸) under pressures up to 33 MPa at SPring-8 beamline BL02B2 (Nishibori *et al.*, 2001 ▸; Kawaguchi *et al.*, 2017 ▸). The sample powder was loaded into an approximately 10 mm length in a fused silica capillary. Acetone (FUJIFILM Wako Pure Chemical, 99% purity) was inserted as the pressure-transmitting medium into the capillary using a micropipette. The maximum applied pressure of 33 MPa in this study is below that reported for the solidification of acetone, *i.e.* more than 1 GPa (Allan *et al.*, 1999 ▸; Talyzin & Luzan, 2010 ▸). The wavelength of the incident X-rays was set to 0.51 Å for the copper measurements. For LN12, the wavelength was set to 0.67 Å to reduce fluorescence X-rays of the Nb atoms. The wavelengths were calibrated with an accuracy of ±0.00001 Å using CeO_2_ standard powder (NIST). The X-ray beam was collimated to 0.5 mm height and 2.0 mm width. Diffraction data were measured for copper in the 2θ range up to 78° with an angular resolution of 0.006° using the double-step mode of multiple Mythen detectors with a sample-to-detector distance of 477.46 mm (Kawaguchi *et al.*, 2017 ▸). The two-dimensional diffraction patterns were also monitored using a flat-panel detector. Diffraction data for LN12 were measured in the 2θ range up to 77° with an angular resolution of 0.01° using an imaging plate detector with a sample-to-detector distance of 286.48 mm (Nishibori *et al.*, 2001 ▸). The powder diffraction measurements were performed using a sample oscillation of 40° during an exposure time of 400 s for each pressure condition.

## 3. Results and discussion

### 3.1. Copper under high pressures

**Figure 2:**
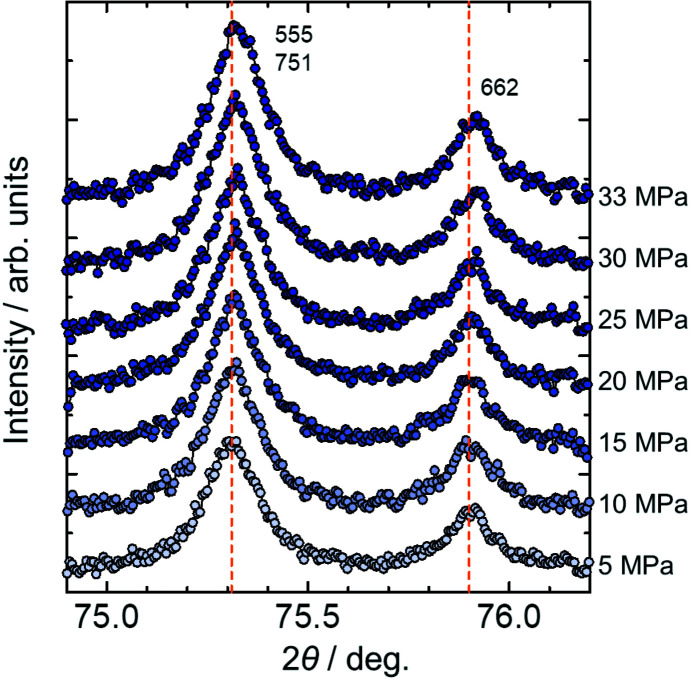
SR-PXRD patterns of copper under high pressures. Acetone was used as a pressure-transmitting medium. The dashed lines show peak positions at 5 MPa.

Fig. 2 ▸ shows the pressure dependence of the powder diffraction data of copper. The two diffraction peaks at 75.3° and 75.9° correspond to the 555 and 751 reflections and the 662 reflection, respectively. Fig. 2 ▸ shows that the peak positions shift to higher angles, *i.e.* lattice contraction, with increasing pressure. The lattice parameters were determined by Rietveld refinement using the *Synchrotron Powder* (*SP*) program (Nishibori *et al.*, 2007 ▸). The fitting results of the Rietveld refinements are shown in Fig. S2. Details of the refinements are also given in the supporting information. A capillary shift of less than 0.01 mm changes the sample-to-detector distance by less than 0.002%, which could affect the estimation of the lattice parameters by an order of 10^−5^ Å. The change of the sample position in the capillary was included in the analysis given in Section S1 and Fig. S3 of the supporting information.

**Figure 3:**
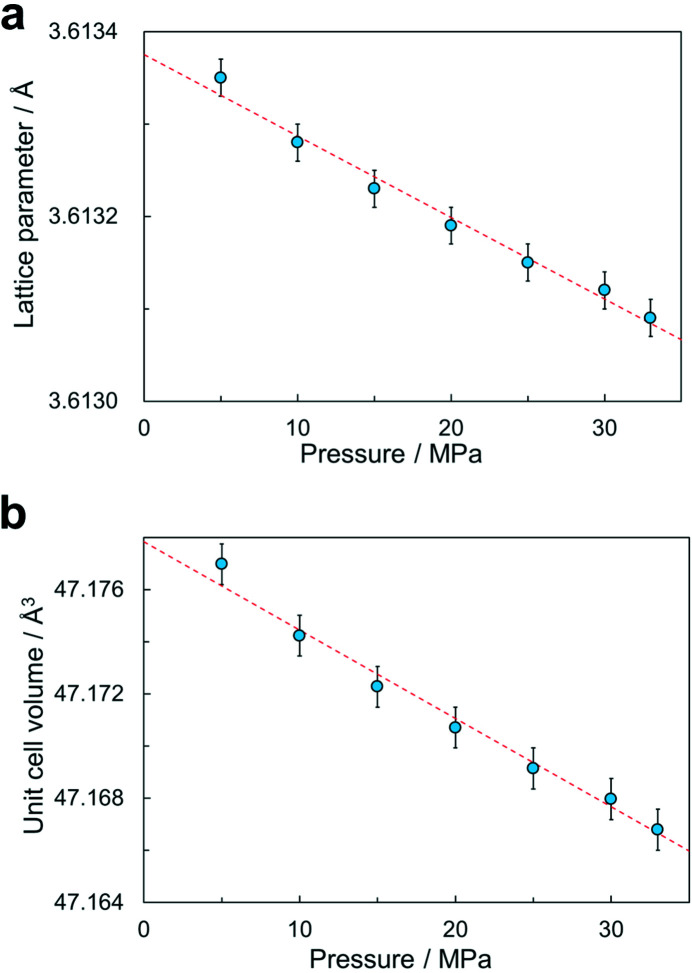
Pressure dependence of the (*a*) lattice parameter and (*b*) unit-cell volume of copper. The dashed lines represent a linear fitting. The error bars for pressure are smaller than the symbols.

Fig. 3 ▸(*a*) shows the determined lattice parameter as a function of pressure. The observed pressure dependence is not affected by the error in the pressure and the shift of the capillary position. In Fig. 3 ▸(*a*), the lattice parameter *a* depends linearly on the pressure *p*. The intercept *a*_0_ of the linear line was 3.613375 (17) Å. The estimated lattice constant for atmospheric pressure agrees with the reported value of 3.6133 Å (Suh *et al.*, 1988 ▸; Huang *et al.*, 2019 ▸). From the linear fitting, the observed compressibility along the crystal axis, −(d*a*/*a*_0_)/d*p*, was 0.0024 (2) GPa^−1^.

Fig. 3 ▸(*b*) shows the pressure dependence of the unit-cell volume. The unit-cell volume, *V*, depends linearly on pressure in the figure. From the linear fitting, the bulk modulus, −*V*d*V*/d*p*, was estimated to be 139 (13) GPa. The observed bulk modulus is consistent with the reported value of 140 GPa (Ledbetter & Naimon, 1974 ▸).

The observed pressure-dependent lattice parameters of copper demonstrated the validity of our developed equipment for atomic-scale observations under high pressure. The pressure range and accuracy of the pressure are appropriate for studies of mechanoresponsive materials (Tu *et al.*, 2017 ▸, 2020 ▸; Yang *et al.*, 2021 ▸). While the error for the obtained bulk modulus is several times larger than that determined in the pressure range of GPa order (Mukaide *et al.*, 2003 ▸; Mishra *et al.*, 2012 ▸), the elastic deformation was monitored quantitatively in the pressure range of 30 times smaller than 1 GPa. Since the bulk modulus depends on pressure due to the anharmonic interatomic potential, it is not obvious that a bulk modulus in the pressure range of MPa order agrees with a zero-pressure bulk modulus determined in the pressure range of GPa order. The bulk modulus of 139 (13) GPa measured in the pressure range from 5 to 33 MPa also confirmed that the reported value (Ledbetter & Naimon, 1974 ▸) describes the elastic property of copper well in the present pressure range.

### 3.2. Repeatable ML material Li_0.12_Na_0.88_NbO_3_:Pr^3+^ under high pressures

**Figure 4:**
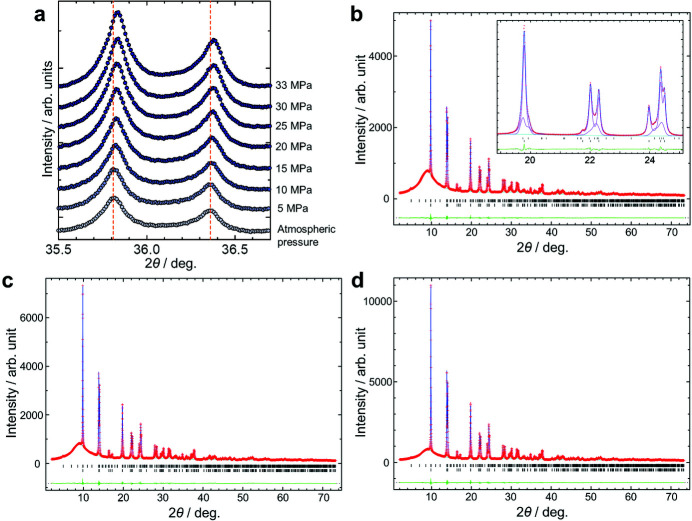
SR-PXRD patterns of LN12 under high pressures applied using acetone as a pressure-transmitting medium. (*a*) Diffraction patterns in the 2θ range from 35.5° to 36.7°. The dashed lines show peak positions at atmospheric pressure. (*b*)–(*d*) Diffraction patterns with fitting result of Rietveld refinement. (*b*) Atmospheric pressure. The inset shows the pattern in the 2θ range from 19° to 25°. Purple lines represent profiles for the *R*3*c* phase and the *Pmc*2_1_ phase. (*c*) 15 MPa. (*d*) 33 MPa. Red points, blue lines, green lines and black bars represent observed, calculated and difference intensities and positions of Bragg reflections, respectively.

Fig. 4 ▸ shows SR-PXRD data of LN12 under high pressures up to 33 MPa. In Fig. 4 ▸(*a*), the intensity of the Bragg reflections increased and the peak positions shifted to a higher angle with increasing pressure. Two phases were observed at atmospheric pressure in Fig. 4 ▸(*b*). These were identified as the trigonal *R*3*c* phase and the orthorhombic *Pmc*2_1_ phase (Hara *et al.*, 2020 ▸). The *R*3*c* phase and the *Pmc*2_1_ phase were observed under applied pressures up to 33 MPa.

Figs. 4 ▸(*b*)–4(*d*) and Fig. S4 also show the fitting result of the Rietveld refinement using the two phases. The details of refinements are given in the supporting information. The reliability factors based on the weighted profile *R*_wp_ and Bragg intensity *R*_I_ were 0.0136 and 0.0203, respectively, for Fig. 4 ▸(*b*). *R*_wp_ and *R*_I_ were 0.0160 and 0.0188, respectively, for Fig. 4 ▸(*c*), and were 0.0182 and 0.0140, respectively, for Fig. 4 ▸(*d*). The scale factor of the *R*3*c* phase was three times larger than that of the *Pmc*2_1_ phase for all of the data. The intensity increase for the Bragg reflections is attributed to the increase of sample amount in the irradiation area by increasing pressure (see the supporting information). Due to the diffraction data shown in Fig. 4 ▸(*b*), the pressure-dependent lattice parameters were difficult to determine for the *Pmc*2_1_ phase.

**Figure 5:**
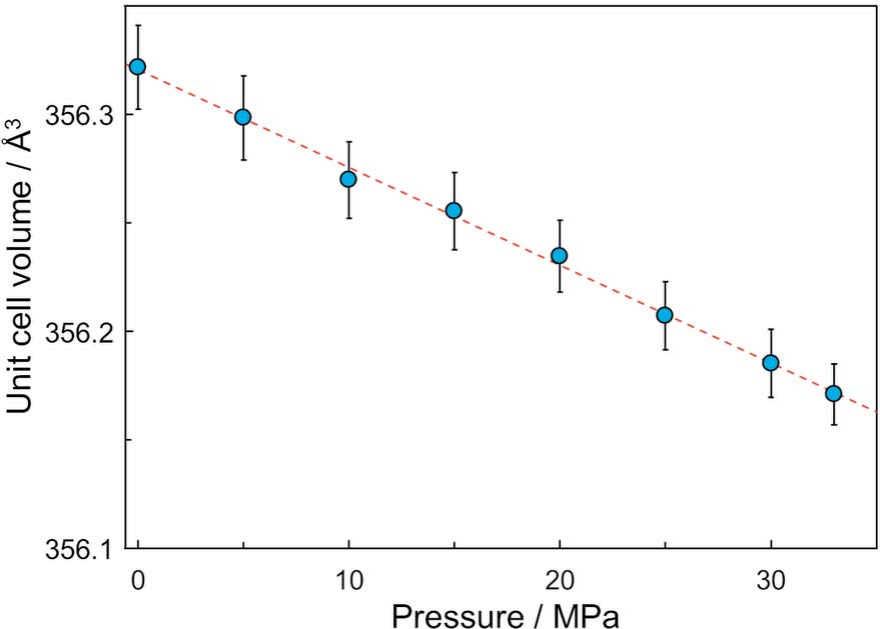
Pressure dependence of the unit-cell volume for the *R*3*c* phase of LN12. The dashed line represents a linear fitting.

Fig. 5 ▸ shows the observed pressure dependence of the unit-cell volume for the *R*3*c* phase. The unit-cell volume depends linearly on pressure in the figure. The bulk modulus was estimated to be 79 (9) GPa for the *R*3*c* phase from the linear fitting. The obtained bulk modulus of LN12 is smaller than the reported values of 117 GPa for the *R*3*c* phase of LiNbO_3_ (Mukaide *et al.*, 2003 ▸) and 157.5 GPa for the *Pbcm* phase of NaNbO_3_ (Mishra *et al.*, 2012 ▸). We speculate that the response to the pressure may be enhanced by an increase of the Na/Li ratio in the *R*3*c* phase and suppressed by the phase transition from *R*3*c* to *Pbcm* owing to these bulk moduli. The speculated elastic behavior matches the reported behavior of the ML intensity (Hara *et al.*, 2020 ▸). A more systematic structural study is required to reveal the structure response of the present mechanoluminescence material.

**Figure 6:**
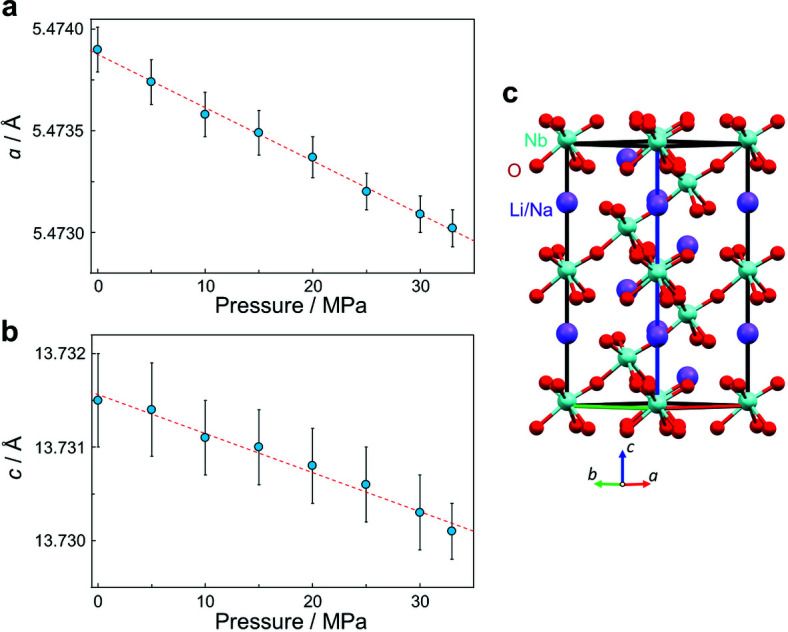
Pressure dependence of the lattice parameters (*a*) *a* and (*b*) *c* for the *R*3*c* phase of LN12. (*c*) Crystal structure of the *R*3*c* phase. Purple, light blue and red spheres represent Li/Na, Nb and O atoms, respectively.

Figs. 6 ▸(*a*) and 6(*b*) show the pressure dependence of the lattice parameters for the *R*3*c* phase. In the figures, the lattice parameters decreased linearly with increasing pressure. From the linear fittings, the compressibility along the *a* and *c* axes was estimated to be 0.0048 (6) GPa^−1^ and 0.0030 (9) GPa^−1^, respectively. The crystal structure of the *R*3*c* phase (Mishra *et al.*, 2015 ▸) is shown in Fig. 6 ▸(*c*). The structure consists of Nb layers and Li/Na layers which are stacking along the *c* axis. The observed anisotropic compressibility shows that the response to pressure was 1.6 (5) times stronger in the layer direction than in the stacking direction.

The observed anisotropic compressibility is important quantitative information toward an understanding of ML. Anisotropy in elasticity plays a role in producing or enhancing an internal electric field in specific directions by applying mechanical force in the proposed ML mechanisms (Feng & Smet, 2018 ▸; Zhang *et al.*, 2019 ▸; Tu *et al.*, 2020 ▸; Yang *et al.*, 2021 ▸). The proposed ML mechanism has not been fully established due to a lack of quantitative understanding. The ML intensity in LiNbO_3_:Pr^3+^ was enhanced by 200% due to a pressure increase of 25 MPa (Tu *et al.*, 2017 ▸). On the other hand, the lattice parameters change by 0.01% due to the pressure increase in LN12. SR-PXRD with our developed equipment can be recognized as a tool for gaining a quantitative understanding of ML materials by quantitative measurement of such small structural changes which enhance the ML properties drastically.

## 4. Conclusions

We have developed equipment for X-ray diffraction experiments under high pressures up to 33 MPa with an accuracy of ±0.1 MPa. In the equipment, a liquid is used as a pressure-transmitting medium. Liquids are good pressure-transmitting media for hydro­static conditions. They also have advantages such as flexibility, reuse by filtering, and ease of handling and leak detection, compared with solid or gas pressure-transmitting media. Since the equipment was developed by modifying a reactor used for *in situ* X-ray diffraction of hydro­thermal synthesis, the temperature can also be easily changed using a heater. We demonstrated the equipment by observing the pressure dependence of the lattice parameters of copper. The bulk modulus, determined as 139 (13) GPa in this study, shows a good agreement with the well known literature value of 140 GPa.

Our developed equipment enables observation of structural changes on the atomic scale of mechanoresponsive materials upon applying a mechanical force, which often ranges from atmospheric pressure to a few tens of MPa for device applications. We applied the method to the repeatable ML material Li_0.12_Na_0.88_NbO_3_:Pr^3+^ (Hara *et al.*, 2020 ▸). The observed anisotropic compressibility of the *R*3*c* phase is useful quantitative information for further studies such as theoretical calculations of the internal electric field to relate the structural change and the ML intensity in order to reveal a repeatable ML mechanism. We believe the advance of atomic-scale observation under high pressures up to a few tens of MPa will reveal the relationship between structure and mechano­responsive properties in detail for atomic-scale design of mechanoresponsive materials.

## 5. Related literature

The following references, not cited in the main body of the paper, have been cited in the supporting information: Lorenzo *et al.* (1995 ▸); Sasaki *et al.* (2018 ▸).

## Supplementary Material

Rietveld refinements of the diffraction data under high pressures. DOI: 10.1107/S160057752300108X/fv5155sup1.pdf
