# Supplementary material for: Synchrotron X-ray powder diffraction under high pressures up to 33 MPa for mechanoresponsive materials
Source: J Synchrotron Radiat. 2023 Mar 10;30(Pt 3):555–60. doi: 10.1107/S160057752300108X (PMC10161880; doi:10.1107/S160057752300108X)
Supplement: Supplementary file 1 [file s-30-00555-sup1.pdf]

JOURNAL OF SYNCHROTRON RADIATION

Volume 30 (2023)

Supporting information for article:

Synchrotron X-ray powder diffraction under high pressures up to 33 MPa for mechanoresponsive materials

Hidetaka Kasai, Jianqiao Liu, Chao-Nan Xu and Eiji Nishibori

S1. Rietveld refinements of the diffraction data under high pressures

The synchrotron radiation (SR) powder X-ray diffraction (PXRD) data were analyzed by Rietveld refinements using synchrotron powder (SP) program (Nishibori *et al*., 2007). The analyzed 2θ ranges were from 13.0° to 76.6° and from 2.0° to 73.3° for the SR-PXRD data for copper and $Li_{0.12}Na_{0.88}NbO_3$:Pr^{3+} (LN12), respectively. The background was described by profile of the capillary, Pearson VII functions and a constant function for the both samples and with thermal diffuse scattering for copper (Sasaki *et al*., 2018). The zero-shift parameters were carefully determined. In Fig. S3, the zero-shift parameters showed a negative value and increased with increasing pressure. The negative zero-shift parameter is attributed to the sample at the bottom of capillary. In the figure, the scale factor increased with increasing pressure, indicating increase of sample amount in the beam irradiation area in the capillary. The observed increase of sample amount is a reason for the increase of zero-shift parameter. The validity of estimated zero-shift parameter was confirmed by the fitting result of Rietveld refinement in Fig. S2 and by agreement with the tendency of scale factor in Fig. S3. The error of lattice parameter was estimated as 2×10^{-5} Å and the order of 10^{-4} Å for copper and LN12, respectively, by refinements with changing a zero-shift parameter. For LN12, the micro amount of doped Pr^{3+}, which was reported to occupy the Li/Na site (Lorenzo *et al*., 1995), was not included in the refinements.

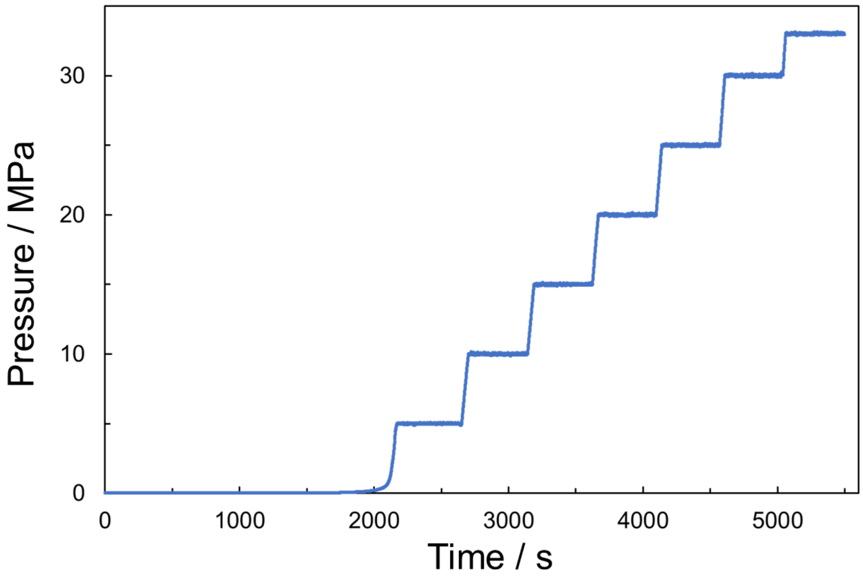

Figure S1 Pressure recorded before, during and after SR-PXRD measurements of LN12.

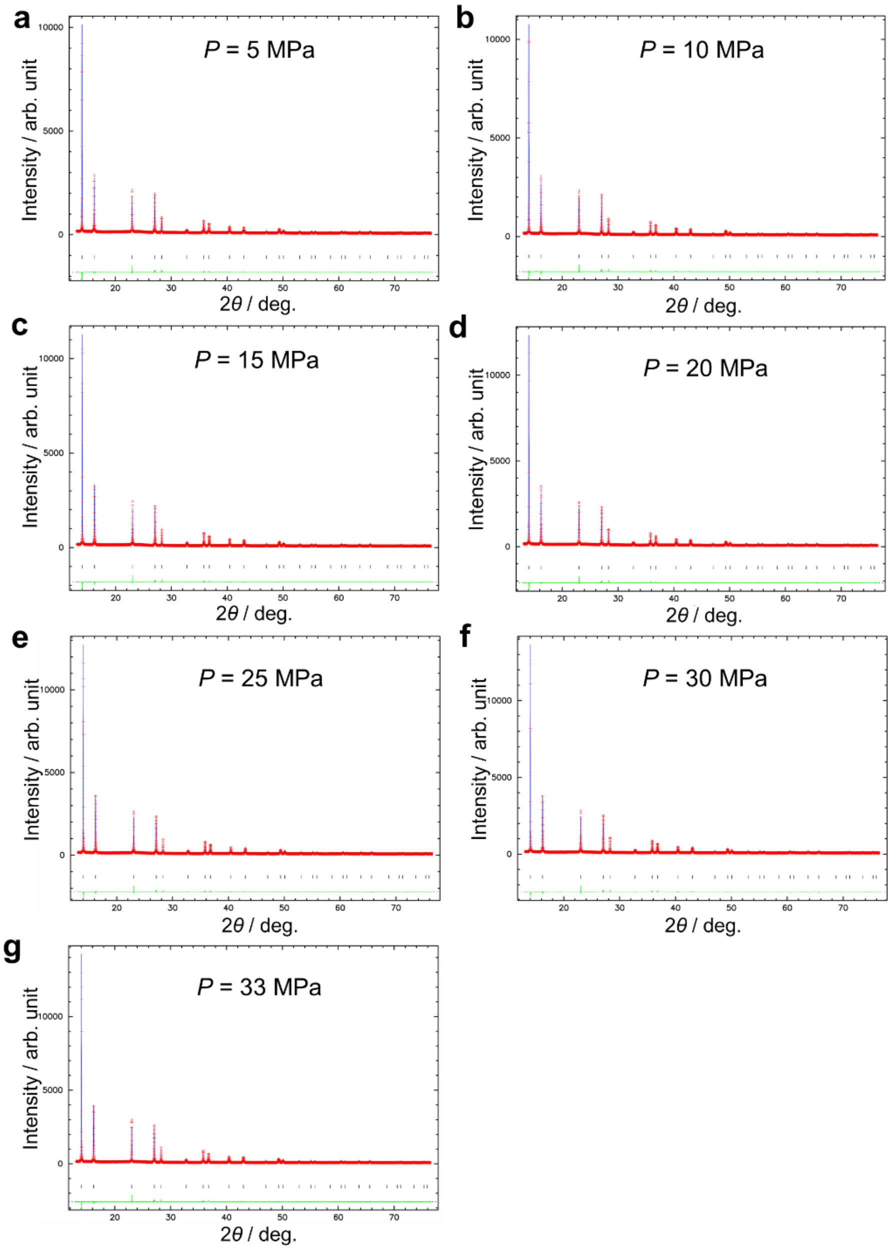

Figure S2 Fitting result of Rietveld refinements for copper under high pressures. (a) R_{wp} = 3.62% and R_I = 6.24%. (b) R_{wp} = 3.64% and R_I = 6.09%. (c) R_{wp} = 3.64% and R_I = 5.70%. (d) R_{wp} = 3.67% and R_I = 5.40%. (e) R_{wp} = 3.72% and R_I = 5.40%. (f) R_{wp} = 4.00% and R_I = 5.73%. (g) R_{wp} = 4.02% and R_I = 5.76%.

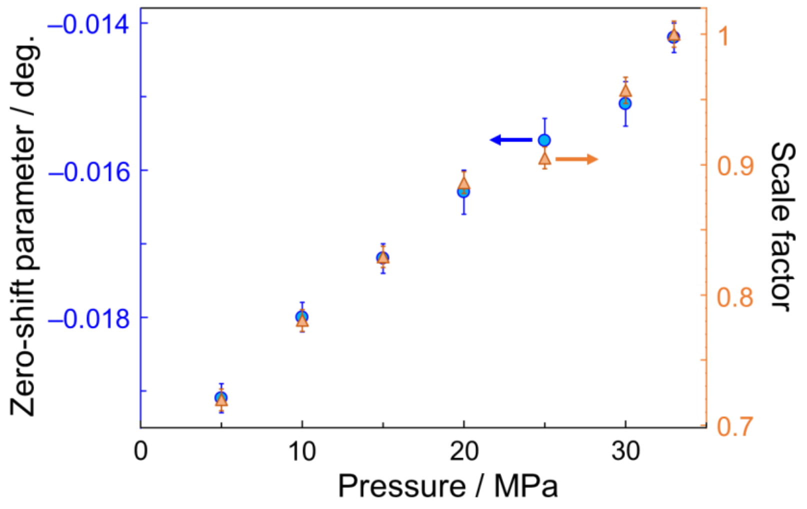

Figure S3 Zero-shift parameter and scale factor of Rietveld refinement for copper. The scale factors are normalized by that for 33 MPa.

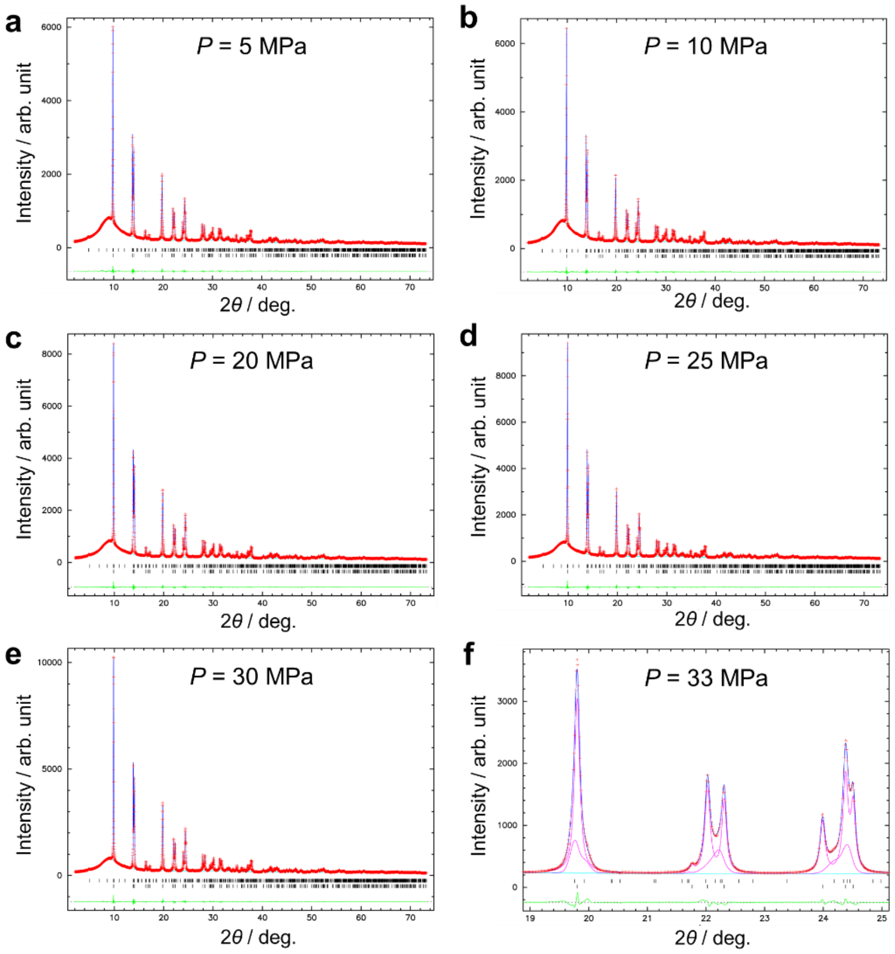

Figure S4 Fitting result of Rietveld refinements for LN12 under high pressures. (a) R_{wp} = 1.44% and R_I = 1.86%. (b) R_{wp} = 1.50% and R_I = 1.77%. (c) R_{wp} = 1.68% and R_I = 1.79%. (d) R_{wp} = 1.77% and R_I = 1.84%. (e) R_{wp} = 1.86% and R_I = 1.69%. (f) 2θ range from 19° to 25°.

References

Lorenzo, A., Jaffrezic, H., Roux, B., Boulon, G. & García-Solé, J. (1995). *Appl. Phys. Lett.* **67**, 3735−3737.

Nishibori, E., Sunaoshi, E., Yoshida, A., Aoyagi, S., Kato, K., Takata, M. & Sakata, M. (2007). *Acta Crystallogr., A: Found. Crystallogr.* **63**, 43−52.

Sasaki, T., Kasai, H. & Nishibori, E. (2018). *Sci. Rep.* **8**, 11964.
